# Depletion and activation of mucosal CD4 T cells in HIV infected women with HPV-associated lesions of the cervix uteri

**DOI:** 10.1371/journal.pone.0240154

**Published:** 2020-10-02

**Authors:** Wilbert Mbuya, Ruby Mcharo, Jacklina Mhizde, Jonathan Mnkai, Anifrid Mahenge, Maria Mwakatima, Wolfram Mwalongo, Nhamo Chiwerengo, Michael Hölscher, Tessa Lennemann, Elmar Saathoff, France Rwegoshora, Liset Torres, Arne Kroidl, Christof Geldmacher, Kathrin Held, Mkunde Chachage

**Affiliations:** 1 National Institute for Medical Research–Mbeya Medical Research Centre (NIMR-MMRC), Mbeya, Tanzania; 2 University of Dar es Salaam -Mbeya College of Health and Allied Sciences (UDSM-MCHAS), Mbeya, Tanzania; 3 Division of Infectious Diseases and Tropical Medicine, University Hospital, LMU Munich, Munich, Germany; 4 German Center for Infection Research (DZIF), Partner site Munich, Munich, Germany; 5 Mbeya Zonal Referral Hospital, Mbeya, Tanzania; University of Pittsburgh, UNITED STATES

## Abstract

**Background:**

The burden of HPV-associated premalignant and malignant cervical lesions remains high in HIV+ women even under ART treatment. In order to identify possible underlying pathophysiologic mechanisms, we studied activation and HIV co-receptor expression in cervical T-cell populations in relation to HIV, HPV and cervical lesion status.

**Methods:**

Cervical cytobrush (n = 468: 253 HIV- and 215 HIV+; 71% on ART) and blood (in a subset of 39 women) was collected from women in Mbeya, Tanzania. Clinical data on HIV and HPV infection, as well as ART status was collected. T cell populations were characterized using multiparametric flow cytometry-based on their expression of markers for cellular activation (HLA-DR), and memory (CD45RO), as well as HIV co-receptors (CCR5, α_4_β_7_).

**Results:**

Cervical and blood T cells differed significantly, with higher frequencies of T cells expressing CD45RO, as well as the HIV co-receptors CCR5 and α_4_β_7_ in the cervical mucosa. The skewed CD4/CD8 T cell ratio in blood of HIV+ women was mirrored in the cervical mucosa and HPV co-infection was linked to lower levels of mucosal CD4 T cells in HIV+ women (%median: 22 vs 32; p = 0.04). In addition, HIV and HPV infection, and especially HPV-associated cervical lesions were linked to significantly higher frequencies of HLA-DR+ CD4 and CD8 T cells (p-values < 0.05). Interestingly, HPV infection did not significantly alter frequencies of CCR5+ or α_4_β_7_+ CD4 T cells.

**Conclusion:**

The increased proportion of activated cervical T cells associated with HPV and HIV infection, as well as HPV-associated lesions, together with the HIV-induced depletion of cervical CD4 T cells, may increase the risk for HPV infection, associated premalignant lesions and cancer in HIV+ women. Further, high levels of activated CD4 T cells associated with HPV and HPV-associated lesions could contribute to a higher susceptibility to HIV in HPV infected women.

## Introduction

Human Immunodeficiency Virus (HIV) and Human Papilloma Viruses (HPV) are both sexually transmitted viruses that cause chronic infections and disease [1,2]. HPVs are a family of about 200 types of small, non-enveloped double-stranded DNA viruses which infect epithelial cells [3]. Depending on their oncogenic propensity, HPV group into either low-risk HPV (LR-HPV) or high-risk HPV (HR-HPV).HR-HPV types HPV16 and HPV18 together are linked to over 70% of all cervical cancer (CC) cases worldwide [4]. Persistent HR-HPV genital infections increase the risk of CC [5]. Most infections with HPV are subclinical; however, immunocompromised individuals, such as HIV+ women, have high incidence and persistent HPV infection and progress rapidly from HPV-associated lesions to invasive CC [6–8]. Antiretroviral therapy (ART) inhibits HIV viral replication, reconstitutes CD4 T-cell counts and immunity, thereby decreasing the risk for opportunistic infections such as Kaposi’s sarcoma, candidiasis and tuberculosis [9,10]. In contrast to most AIDS-defining diseases, the burden of HPV-associated premalignant and malignant lesions remains high in HIV infected individuals despite the initiation of ART [7].

It has been shown that the frequency of immune cells and inflammatory cytokines within HPV-associated premalignant cervical lesions are suppressed in HIV infected women [10–12], demonstrating a direct effect of HIV on the cervical immune response. Furthermore, chronic HIV-induced immune activation, marked by an increased proportion of CD38+ and HLA-DR+ systemic T cells [13], is associated with immune dysfunction and damage of mucosa [14–18], resulting in an increased persistence of HPV infections in HIV+ individuals [19,20]. Upregulation of HLA-DR has also been reported on epithelial cells in pre-cancerous lesions and genital warts [21].

Sexually transmitted infections such as gonorrhoea, syphilis, and chlamydia are known to cause genital ulcers and trigger an inflammatory response that is associated with recruitment of immune cells, including CD4 T cells, to the genital area, thereby increasing the risk for HIV acquisition [22,23]. An increased risk for HIV acquisition has also been reported in HPV infected women, especially for those with multiple HR-HPV infections [24–28]. This might be due to mucosal and cytokine milieu changes induced by HPV infection or recruitment of activated CD4 T cells to the cervix; as such cells provide suitable targets for HIV infection and replication. The inflammation associated with lesions has been shown to increase the HIV acquisition risk [28–32].

To elucidate the mechanism by which HIV increases the risk for HPV-infection or progression of associated lesions and vice versa, we analysed the frequency of T-cell lineage, memory, and activation markers, as well as the HIV co-receptors CCR5 and α_4_β_7_ within peripheral and mucosal T cells by flow cytometry. Characterisation of T cells in the cervical mucosal is crucial to understand the impact of HPV, an epitheliotropic virus without a systemic phase, on the cervical immune response [33–35]. Specifically, we studied phenotypic differences in systemic and mucosal T-cell populations and the effect of HPV and HIV infection on the T-cell composition in the cervix. We further sought to understand, whether HPV infection, as well as disease, is associated with cervical immune activation and alterations in the expression of HIV co-receptors, thereby rendering HPV infected women at risk higher of acquiring HIV.

## Material and methods

### Study population

Volunteers were recruited as part of an HIV-HPV (2H) study that started in 2013 and is currently ongoing in Mbeya, Tanzania. The 2H study is a prospective, longitudinal case-control study which aims to assess the effect of HIV infection and ART treatment on HPV infection and disease. For the data presented herein, 468 HIV+ and HIV- women from 18 years of age and above attending the cervical cancer screening clinics in Mbeya were recruited. Only cytobrush samples with complete laboratory documentation that could be linked to clinical data, no visible blood contamination, more than 150 T cells events in the CD3 gate, and satisfactory staining quality were included in the statistical analysis. Of these 468 samples, cervical pathology and HPV genotyping data were available for 440 and 213 women respectively, while data for ART usage was available for 201 of the 215 HIV+ women.

### Ethical consideration

All study participants were fully briefed on the study, and written informed consent was obtained prior to enrolment/participation. The Mbeya Medical Research and Ethics Review Committee reference: MRH/R.10/8/Vol. VI/107, the Tanzanian National Health Research Ethics Committee reference: NIMR/HQ/R.8a/Vol. IX/1422 and the Ethics Committee of the medical faculty of the University of Munich (Project ID: 308–11) provided specific approval for this study before commencement of the study.

### Specimen collection

Cervical cells were obtained from the endocervix by inserting a cytobrush (Solann) into the endocervical wall and gently rotating the brush 360°. Part of the specimen was used for cytological examination by Papanicolaou testing with the remainder being collected in 5 mL complete media (10% FBS (Sigma) in RPMI-1640 (Gibco), 50U/mL Penicillin, 50μg/mL Streptomycin (Gibco) and 1x antibiotic-antimycotic solution (Sigma)). A second cytobrush sample was obtained for HPV genotyping and stored in 5 mL PreservCyt cell collection media (Roche). Cells were thoroughly flushed from the cytobrush using a Pasteur pipette and complete media. Cervical cell suspension from the first cytobrush was then transferred to a 5 mL falcon tube, pelleted by centrifugation at 570*g* for 10 minutes, washed with 2ml wash buffer (PBS with 1% bovine serum albumin and 0.05% sodium azide) and centrifuged (1600rm for 6 minutes) twice before flow cytometric analysis. Cervical cells from the second cytobrush were aliquoted and short-term (-20°C) or long-term (-80°C) stored for HPV genotyping.

EDTA anti-coagulated peripheral blood was collected by venepuncture, and whole blood used for *ex vivo* analysis. Both specimen types, cervical cells and whole blood, were processed immediately after specimen collection.

#### Flow cytometric ex vivo characterisation of cervical and peripheral T-cell populations

The following anti-human antibodies were used for staining of cytobrush and whole blood samples: CD3-Pacific Blue (clone UCHT1, BD), CD4-PerCP Cy5.5 (clone OKT4, eBioscience), CD8-Horizon-V500 (clone RPA-T, BD), CD45-APC-H7 (clone 2D1, BD), CD45RO-PE (clone UCHL1, BD), HLA-DR-APC (cloneG46-6 BD-Pharmingen) (only for cytobrush samples), α4β7-FITC (clone FIB504, Biolegend), and CCR5-PEcy7 (clone 2D7/CCR5, BD). Cells were incubated with the antibody cocktail at 4°C for 30 minutes in the dark, washed with FACS wash buffer (PBS with 1% bovine serum albumin and 0.05% sodium azide), and acquired on a FACS Canto II (BD) after fixation with 2% paraformaldehyde in water. Only cervical cytobrush samples without visible red blood cells, satisfactory staining quality, and T-cell counts above 150 cells were included in the analysis. For whole blood, a cut-off of at least 10,000 lymphocyte events was applied. Fluorescent spill over compensation was conducted with antibody capture beads (BD) stained separately with the individual antibodies used in the test samples. Gating was guided for both sample types (peripheral blood and cytobrush) separately by fluorescence minus one (FMO) controls. Flow cytometry data was processed using FlowJo version 10.4 (Tree Star Inc.).

### Genotyping of cervical HPV infection

To identify the infecting HPV genotype, DNA was extracted from cervical cells stored at -20°C in PreservCyt Solution (Roche) using QIAamp DNA mini kit (Qiagen), followed by HPV genotyping using the LINEAR ARRAY® HPV Genotyping Test (Roche) as per manufacturer’s instructions. This assay qualitatively detects and identifies thirty-seven HPV genotypes (6, 11, 16, 18, 26, 31, 33, 35, 39, 40, 42, 45, 51, 52, 53, 54, 55, 56, 58, 59, 61, 62, 64, 66, 67, 68, 69, 70, 71, 72, 73, 81, 82, 83, 84, IS39 and CP6108). Women who tested positive for any of the 37 HPV types detected by the Roche linear array genotyping test were considered to have an ongoing cervical HPV infection. Only the presence or absence of an ongoing HPV infection, irrespective of infecting subtype, was considered for the following analysis.

### Evaluation of histological and cytological pathology

Routine cytology by Papanicolaou testing and, if biopsied were collected, histology based on Haematoxylin & Eosin staining were performed at the pathology department of the Mbeya Zonal Referral Hospital (MZRH) Pathology department. The lesions were diagnosed and reported as per the Bethesda system for cytopathological classification.

### CD4 cell counts

Absolute CD4 T-cell counts were analysed from blood samples as part of routine patient management using BD Trucount tubes (BD) and were acquired on a BD FACSCalibur (BD).

### Statistical analysis

Baseline characteristics, as well as clinical and HIV related information were extracted from data collection forms that were entered and quality controlled in a study tailored SQL database. Statistical testing included the following tests; Mann-Whitney U test was employed to assess the effect of HIV and/or HPV infection on the proportion of CD4+ and CD8+ T cells, as well as the frequency of α_4_β_7_, CCR5 and HLA-DR expression on T cells with respect to HIV and or HPV infection. Wilcoxon matched-pairs signed ranks test was used to evaluate the difference in proportions of CD4, CD8, HIV receptors CCR5 and α_4_β_7_ and the memory marker CD45RO between peripheral blood and cervical mucosal sample from the same participant. Spearman’s rank order correlation test was used to evaluate the association between HLA-DR on CD4+ T cells with the corresponding CD4 count of the same participant. The sample sizes for different analysis may differ because not all data for all parameters was available for each patient. The exact number is indicated in the respective figure legends. For all tests a two-tailed p-value of < 0.05 was regarded as significant. Stata version 14 (StataCorp, USA) and GraphPad Prism software version 7 (GraphPad Software Inc, USA) were used for statistical analysis.

## Results

### Description of the cohort

All women included in this study were attending the cervical cancer screening clinics in Mbeya, Tanzania and were enrolled into the 2H study between 2013 and 2017. **Table 1** provides clinical details of the study volunteers. Overall, 468 women were included in this analysis, of which 215 (46%) were HIV+. ART status was recorded for 201 of these HIV+ women with 153 (71%) being on ART treatment. A definitive pathological diagnosis was available for 440 women; 15 women had cytologically confirmed Low-grade Squamous Intraepithelial Lesions (LSIL), 13 were classified as High-grade Squamous Intraepithelial Lesions (HSIL), 32 were diagnosed with cervical cancer, and 380 had no lesions. HPV genotyping data was available for 213 women of which 57% (n = 122) had HPV infection(s).

**Table 1:** Clinical details of study participants included in the cohort stratified by HIV status. The number of study participants (n) is given for each strata.

Clinical parameter	Total N = 468	HIV—N = 253	HIV + N = 215
**Median age [IQR]**	38 [31–45]	38 [29–48]	38 [31–43]
**HIV+ on ART**	n/a	n/a	153 (71%)
**HIV+ not on ART**			48 (22%)
**Missing ART information**			14 (7%)
**Median CD4 counts [IQR (cells/μl)]**	n/a	n/a	422 [245–614]
**Pathological diagnosis†**	**N = 440**	**N = 240**	**N = 200**
**CC**	32 (7%)	16 (7%)	16 (8%)
**HSIL**	13 (3%)	6 (3%)	7 (4%)
**LSIL**	15 (3%)	5 (2%)	10 (5%)
**No lesion**	380 (86%)	213 (89%)	167 (84%)
**Cervical HPV diagnosis**	**N = 213**	**N = 113**	**N = 100**
**HPV infected**	122 (57%)	47 (42%)	75 (75%)

† pathology diagnosis based on cytology and confirmed by histology

CC = cervical cancer, HSIL = high grade intraepithelial lesion, LSIL = low grade intraepithelial lesion, ART = antiretroviral therapy

### Cervical T cells differ from peripheral blood T cells

**Fig 1:**
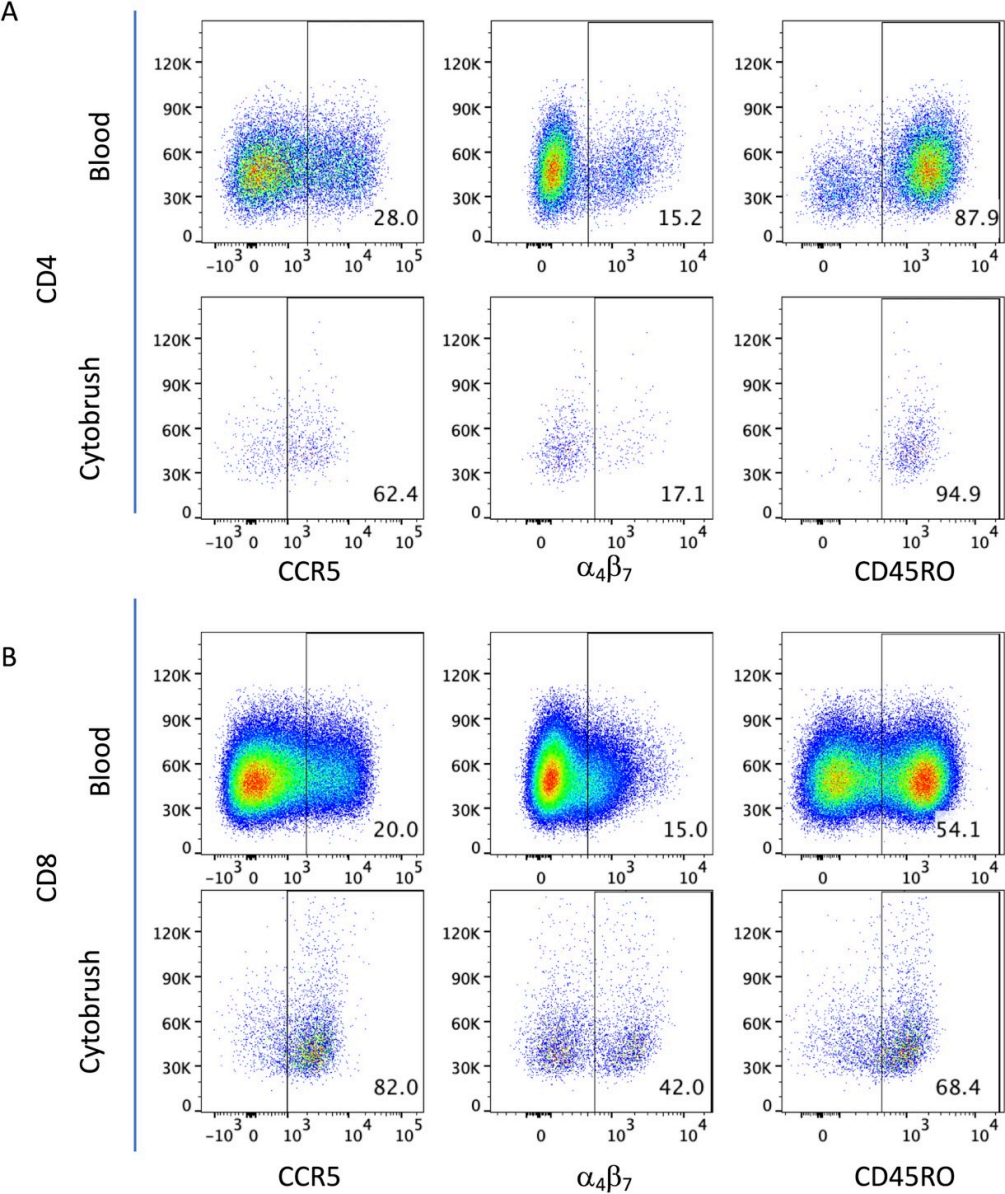
Phenotypic characterization of T cells from cervical mucosa and peripheral blood. Representative pseudocolour dot plots of peripheral whole blood and cervical cells showing the expression of CCR5 (left panels), α_4_β_7_ (middle panels) and CD45RO (right panels) on CD4 and CD8 T cells (**A** and **B**, respectively). Samples were first gated on CD3+CD45+ T cells and then analysed for the expression of phenotypic markers of interest on CD4+ and CD8+ T cells. FMO controls were used to set gates for each sample type (peripheral blood or cytobrush) separately.

To assess compositional and phenotypic differences between T cells in the cervical mucosal and the peripheral blood compartment, we first compared CD4 and CD8 T cells from both anatomical compartments in a subgroup of 39 women (22 HIV- & 17 HIV+). As memory CD4 T cells are the primary target for HIV infection and replication we also compared the percentage of memory T cells as defined by CD45R0 and HIV co-receptor (CCR5 and α_4_β_7_ integrin) expression. Representative flow cytometry dot plots from one participant for these markers are shown in **Fig 1** and the overall results are presented in **Table 2**. The median percentage of CD3+CD45+ cells of total events was much lower in the cervical cells than in peripheral blood (0.33% vs 11.71%; p < 0.001) (**S1A Fig**). A skewed CD4/CD8 ratio that was observed in blood following HIV infection was mirrored in the mucosal samples. Interestingly, CD4 median fluorescence intensity (MFI) was 1.8-fold higher in peripheral blood than in cervical cells, while CD4+CCR5 and CD8 MFI were 1.2 and 2.6 fold higher in cervical cells than blood, respectively (both p<0.001). CD45RO+ memory T cell frequencies were 1.21 and 1.74 fold higher in cervical than in peripheral blood CD4 and CD8 T cells (p = 0.011 and p < 0.001, respectively). Percentages of cells positive for the HIV co-receptor CCR5 were over 2-fold increase in the cervical mucosa compared to peripheral blood (p<0.001), both, on total and memory CD4 T cells. However, the percentage of α_4_β_7_-integrin positive cells within total and memory CD4 T cells was comparable between the two compartments. Median frequencies of CCR5+ or α_4_β_7_+ CD8 T cells were higher in the mucosa than in the blood (p < 0.001 and p = 0.002, respectively). Stratification of data by HIV status did not alter the observed difference in relative proportions of mucosal and peripheral CD45RO+ and CCR5+ T cells. Stratification by HIV status did not reveal an apparent effect of HIV on the relative proportion of CD45RO+ and CCR5+ mucosal and peripheral T cells. However, among HIV+ subjects, mucosal samples tended to have reduced frequencies of α_4_β_7_+ CD4 and CD8 T cells compared to blood (% median: 21.50 vs 16.30 and % median: 39.10 vs 30.80, p = 0.525 and respectively, **S1A and S1B Fig**).

**Table 2:** Comparison of HIV co-receptors (CCR5 and α_4_β_7_) and memory marker (CD45RO) expression on T cells in mucosal cytobrush samples vs peripheral blood from HIV positive and negative women (n = 39; 22 HIV- & 17 HIV+).

parameter	parent population	cervical mucosa [median]	peripheral blood [median]	p-value
**% CD4+**	CD45+CD3+ T cells	41.9	50.3	0.178
**% CD4+ (HIV-)**	CD45+CD3+ T cells	54.1	60.3	0.147
**% CD4+ (HIV+)**	CD45+CD3+ T cells	23.9	24.2	0.384
**CD4/CD8 ratio (HIV-)**	CD45+CD3+ T cells	1.6	2	0.384
**CD4/CD8 ratio (HIV+)**	CD45+CD3+ T cells	0.4	0.4	0.525
**CD4 MFI**	CD45+CD3+ T cells	1788	3332	<0.001
**% α_4_β_7_+**	CD45+CD3+CD4+ T cells	20.6	17.8	0.646
**% α_4_β_7_ MFI**	CD45+CD3+CD4+ T cells	1204	1152	0.686
**% CCR5+**	CD45+CD3+CD4+ T cells	46.3	18.2	<0.001
**CCR5+ MFI**	CD45+CD3+CD4+ T cells	1957	2442	<0.001
**% CD45RO+**	CD45+CD3+CD4+ T cells	78.8	64.6	0.011
**% α_4_β_7_+**	CD45+CD3+CD4+CD45RO+T cells	20.4	19.1	0.618
**% CCR5+**	CD45+CD3+CD4+CD45RO+ T cells	49.1	23.9	<0.001
**% CD8+**	CD45+CD3+ T cells	44.8	38.6	0.666
**CD8+ MFI**	CD45+CD3+ T cells	5313	1994	<0.001
**% α_4_β_7_+**	CD45+CD3+CD8+ T cells	46.8	32.7	0.002
**α_4_β_7_+ MFI**	CD45+CD3+CD8+ T cells	1292	849	<0.001
**% CCR5+**	CD45+CD3+CD8+ T cells	65.6	28.8	<0.001
**CCR5+ MFI**	CD45+CD3+CD8+ T cells	2288	2283	0.7562
**% CD45RO+**	CD45+CD3+CD8+ T cells	43.6	25	<0.001

Overall, these results show that frequencies of HIV co-receptor positive T cells differ significantly between peripheral blood and cervical mucosa T cells.

### HPV infection is associated with decreased frequencies of CD4 T cells in the cervix of HIV+ women

**Fig 2:**
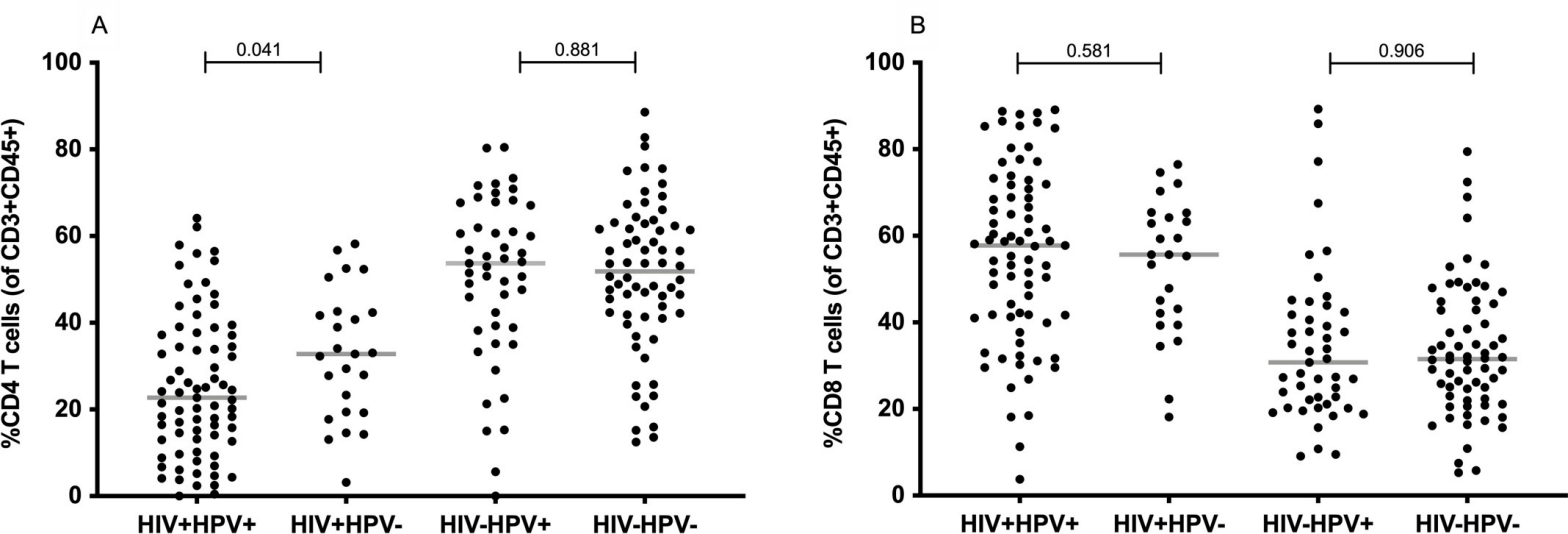
Cervical CD4 and CD8 T-cell frequencies stratified by HIV and HPV infection status (n = 213; HIV+ HPV+ = 75, HIV+ HPV- = 25, HIV- HPV+ = 47, HIV- HPV- = 66). Frequency of cervical CD4 (**A**) and CD8 (**B**) T cells is shown as percent of CD3+CD45+ lymphocytes. Each dot represents one patient. HPV and HIV infections status are indicated on the x-axis. P-values were calculated using the Mann-Whitney U-test. For this graph and the subsequent graphs, the median is indicated by a horizontal line within the data points while the p-values are indicated on top of the data points.

Next, we assessed the effect of an infection with HIV, HPV, or a co-infection with both viruses on mucosal T-cell populations of all women with available HPV genotyping results (n = 213). As expected, HIV infection was significantly associated with lower CD4 and higher CD8 T cell frequencies in the cervix (median % in the total cohort (HIV- vs HIV+): 50.5 vs 25.1 for CD4 and 32.1 vs 55.7 for CD8; p-value for both < 0.001, (**S2A and S2B Fig**)), similar to what is commonly described in peripheral blood. Antiretroviral treatment increased CD4 T cell frequencies in the cervix, but not to levels observed in HIV- individuals (median % (ART- vs ART+): 16.3 vs 26.8, p = 0.001, **S3A Fig**). When stratified by both viral infections, a further moderate decline in median frequency of cervical CD4 T cells was observed in HIV+HPV+ women compared to HIV+HPV- women (median %: 22.7 vs 32.8, p = 0.041). In contrast, amongst HIV- women, HPV infection had no apparent influence on CD4 or CD8 T cell frequencies (**Fig 2A**). No difference can be seen in the proportion of CD8 T cells between HIV+ women with or without HPV (**Fig 2B**).

### HPV infection does not alter the frequency of CD4 T cells expressing CCR5 and α_4_β_7_ in the cervix

**Fig 3:**
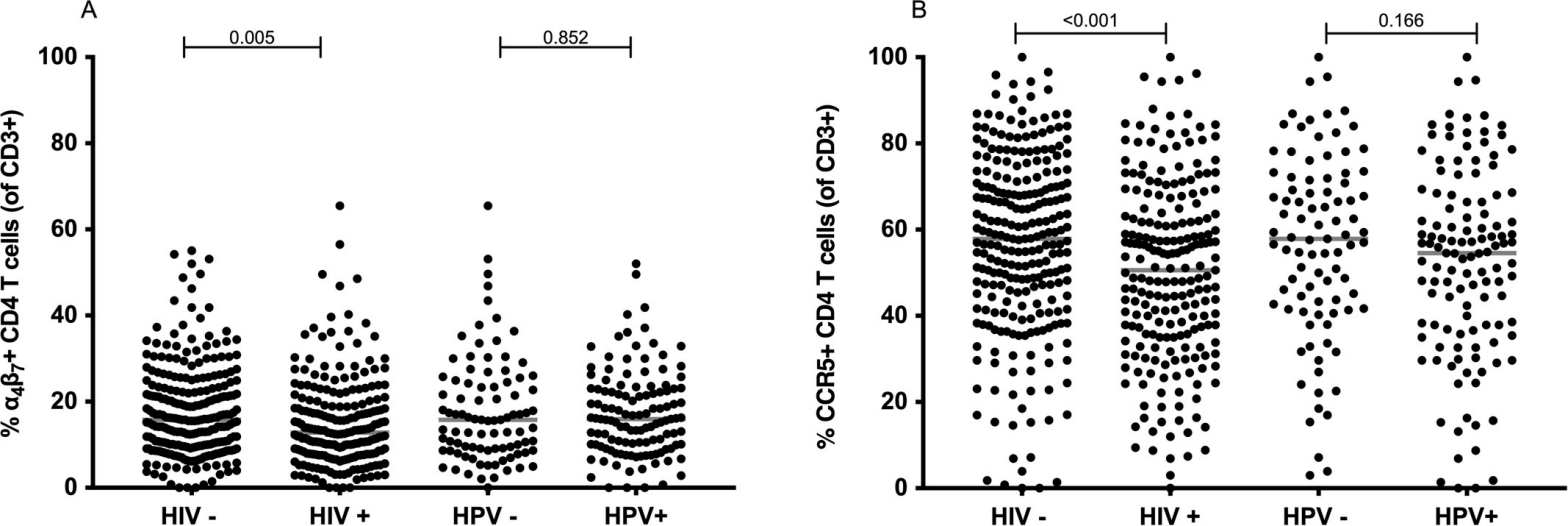
α_4_β_7_ and CCR5 frequencies on cervical CD4 T cells stratified by HIV and HPV infection status (HIV- = 215, HIV+ = 253, HPV— = 91, HPV+ = 122). The frequency of α_4_β_7_+CD4 (**A**) and CCR5+CD4 (**B**) T cells is shown as a proportion of CD3 T cells for each sample. HIV and HPV infections status is indicated on the x-axis. Median frequencies are indicated. Statistical analysis was performed using the Mann-Whitney U-test.

Since CCR5 and α_4_β_7_ are essential for HIV to infect CD4 T cells, we assessed whether HPV infection alters the expression of these receptors on cervical T cells, thereby rendering HPV infected individuals more prone to HIV infection. HPV infection was not associated with a significant change in frequencies of α_4_β_7_+ CD4 (p = 0.852) and CCR5+ CD4 (p = 0.166) cervical T cells (**Fig 3**). The frequency of these receptors on CD4 T cells was not significantly different when the data was further stratified into different permutations of HIV and HPV co-infections (**S4A and S4B Fig**). On the other hand, in HIV+ women we observed significantly lower frequencies of CD4 T cells expressing α_4_β_7_ (p = 0.005) or CCR5 (p < 0.001) as compared to HIV- women, irrespective of HPV infection (**Fig 3**). Among these HIV+ women, frequencies of CCR5 and α_4_β_7_ on CD4 T cells remained at similar levels despite ART treatment (**S3B and S3C Fig, Fig 3**)

### HPV infection, HPV-associated lesions and HIV are linked to an increased frequency of HLA-DR+ T cells in the cervix

**Fig 4:**
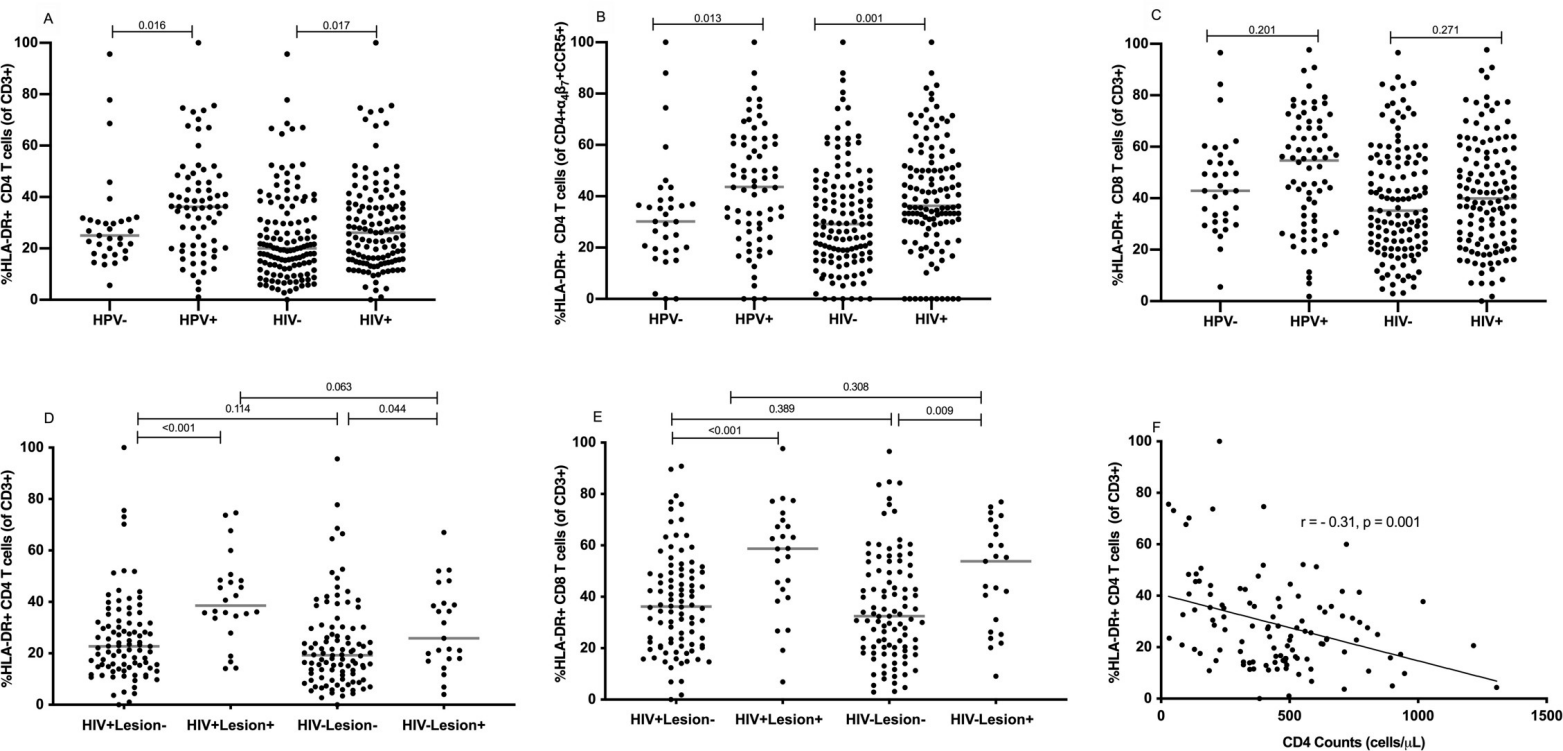
Effect of HIV, HPV and HPV-associated pathology on frequencies of HLA-DR+ T cells. **A, B, C**: Comparison of the frequencies of HLA-DR+ CD4 (**A**), HLA-DR+CCR5+ α_4_β_7_+ CD4 (**B**) and HLA-DR+ CD8 (**C**) T cells on HIV- (n = 129) vs HIV+ (n = 126) women and HPV- (n = 33) vs HPV+ (n = 70). The frequency of HLA-DR+ cells is shown as percent of CD4, CCR5+ α_4_β_7_+ CD4 or CD8 T cells in relation to HIV and HPV status. **D**,**E**: Comparison of the percentage of HLA-DR+ cells in CD4 (**D**) and CD8 (**E**) T cells in relation to different permutations of HIV and cervical lesion (n = 237; HIV+ Lesion- = 92, HIV+ Lesion+ = 24, HIV- Lesion- = 98, HIV- Lesion+ = 23). **F**: Negative correlation of HLA-DR+CD4+ cervical T cell frequencies with peripheral blood absolute CD4 T-cell counts in HIV+ women (n = 113, r = -0.3111, p < 0.001). Median frequencies, correlation coefficient, and p-values are indicated in the graphs. Statistical analyses were performed using Mann-Whitney U-test (**Fig 4A–4E**) and Spearman correlation test (**Fig 4F**).

To determine the effect of HPV infection and HPV-associated lesions (HSIL, LSIL, and CC) on immune activation in cervical T cells, we determined the frequency of T cells expressing the activation marker HLA-DR. The proportion of HLA-DR+ cells on total cervical CD4 T cells and CCR5+ α_4_β_7_+ CD4 T cells was higher in HPV+ compared to HPV- individuals (median % for HLA-DR+ CD4 T cells: 36.20 vs 25.00, p = 0.016; **Fig 4A**; median % CCR5+ α_4_β_7_+ HLA-DR+ CD4 T cells: 43.65 vs 30.20, p = 0.014; **Fig 4B**). However, when the data was stratified by HIV and HPV infections, no statistical difference was observed in the frequency of HLA-DR+ CD4 or HLA-DR+ CD8 T cells between the groups (**S4C and S4D Fig**). Furthermore, HPV-associated lesions were significantly associated with higher percentages of HLA-DR+ T cells, in both CD4 (**Fig 4D**) and CD8 T cells (**Fig 4E**). For CD4 T cells, HIV+ women with lesions had high median percentages of HLA-DR+ cells as compared to HIV+ women without lesions (median %: 39 vs 23, p = <0.001; **Fig 4C**). Similarly, HIV- women with lesions had a significantly higher frequency of HLA-DR+ CD4 T cells (median %:26 vs 19, p = 0.044) than those without lesions (**Fig 4D**). The same effect of lesions on the frequency of HLA-DR+ cells is seen for CD8 T cells, where women with lesions had a higher percentage of cells expressing HLA-DR as compared to those without lesions (HIV+: median %: 36 vs 59, p < 0.001, HIV-: median %: 32 vs 54, p = 0.009; **Fig 4E**).

HIV+ women had moderately higher frequencies of HLA-DR+ CD4 T cells and CCR5+ α_4_β_7_+ HLA-DR+ CD4 T cells when compared to HIV- women (median % for HLA-DR+ CD4+ T cells: 26 vs 20, p = 0.017; median % for CCR5+ α_4_β_7_+ HLA-DR+ CD4 T cells: 36 vs 29, p = 0.001; **Fig 4A and 4B** respectively). No significant effect of an HIV infection on the frequencies of HLA-DR+ CD8 T cells was observed (%median 35 vs 40, p = 0.271; **Fig 4C**). Amongst HIV+ women, no significant difference in the frequency of HLA-DR+ CD4 T cells was observed between women on and off ART (**S3D Fig**). A negative correlation of the percentage of HLA-DR+ CD4+ cervical T cells to peripheral blood absolute CD4 counts of the same individual could be observed in HIV+ women (r = 0.31, p = 0.001; **Fig 4F**).

## Discussion

Women living with HIV, even under ART-treatment, have an increased risk for incident and persistent HPV infection and progress more rapidly to HPV-associated lesions and eventually invasive cervical cancer [6–8]. Immune cells residing in the cervical mucosa are important in the control of HIV and HPV infections [36] and have been shown to be dysregulated in HIV+ women [11,12,37,38]. To analyse whether the high risk for HPV infection and fast progression to HPV-associated cervical cancer in HIV+ women [39] might be associated with HIV-induced changes in cervical T cells, we characterised the expression of HIV co-receptors and activation markers on cervical and peripheral T cells in relation to HIV and HPV infection, and disease, in a cohort of 468 HIV+ and HIV- women attending the cervical cancer screening clinics in Mbeya, Tanzania. We report that the percentage of T cells expressing the memory marker CD45RO as well as the HIV co-receptor CCR5 and α_4_β_7_ in the cervix significantly differs from peripheral blood, underlining the importance of studying the local immune response at the site of HPV-infection. Generally, In healthy individuals, T cells comprise 15–30% of all immune cells in whole blood, CD4 T cells being the majority with 60–70% while CD8 T cell are 20–30% of all T cells [40,41]. The proportion of cervical T cells and associated subsets in health individuals are unknown since the majority of studies report proportions and functionality in these cells during infections and diseases [42–45]. Herein we report a skewed CD4/CD8 T-cell ratio in the blood of HIV+ women was mirrored in the cervical mucosa, and the frequency of CD4 T cells was further decreased in HIV+ women with a co-existing HPV-infection. Low CD4 T-cell frequencies in the cervical mucosa may, therefore, contribute to increased HPV infection and persistence rates in HIV+ women. HIV-infection was also associated with a marked decrease in CCR5+ and α_4_β_7_+ cervical CD4 T cells and an increase in HLA DR^+^ CD4 T cells, demonstrating an apparent effect of HIV infection on the homing properties and activation of cervical T cells. It can, therefore, be speculated that these alterations contribute to a less effective HPV-clearance in HIV+ women.

HPV infection and HPV-associated lesions were also linked to elevated levels of immune activation thus may contribute to immune dysregulation through over-activation in the cervix. Human T cells show a marked differential distribution of cell subsets and phenotypical characteristics in different body compartments [46]. Assessing differences in T-cell marker and HIV co-receptor expression on peripheral and cervical T cells, we observed that the proportion of T cells expressing the HIV co-receptor CCR5 and α_4_β_7_ as well as the memory marker CD45RO is higher in cervical T cells than in peripheral blood T cells. The higher frequency of CCR5 expressing cells in the cervix is in line with the findings of McKinnon et al., [44] who reported a higher frequency of CCR5+ Th17 cells in the cervix compared to blood. Additionally, cervical T cells when compared to peripheral T cells showed higher CD4 molecule expression per cell, as demonstrated by an increase in the MFI. We, therefore, hypothesize that high proportion of CD45RO+CCR5+CD4+ cells together with high cellular CD4 expression density increases HIV susceptibility of mucosal cervical as compared to peripheral blood T cells.

HIV induced systemic loss of CD4 T cells increases susceptibility to different opportunistic infections, including HPV [47,48].Similarly, depletion of CD4 T cells at mucosal sites [49,50] and within the genital tract during HIV infection has previously been demonstrated [45]. In line with these earlier findings, we report a low frequency of cervical CD4 T cells in HIV+ women and further lower CD4 T cell frequencies in HIV+HPV+ compared to HIV+HPV- women. However, among HIV- women, HPV infection was not associated with a change in CD4 T-cell frequencies which implies that the low percentage of CD4 T cells in HIV+HPV+ women is an effect of the HIV rather than the HPV infection. Our results are in line with a study by Palesfsky *et al*., demonstrating HIV+ women with low CD4 T-cell counts were more likely to be HPV infected [48]. A low frequency of cervical CD4 T cells is likely to be associated with immune dysfunction and elevates the risk of acquiring an HPV infection.

Both CCR5 and α_4_β_7_ are essential for HIV attachment to CD4 T cells. It is well established that HIV preferentially infects CD4 T cells expressing CCR5 [51,52] or α_4_β_7_ [53,54]. McKinnon *et al*., observed an enhanced in-vitro HIV susceptibility in cervical CD4 T cells of HIV -women was associated with co-expression of CCR5 and α_4_β_7_ [55,56]. However, their study did not address whether these markers are altered in HPV-infected individuals. Our results show that HPV infection has no effect on the percentage of cervical CD4 T cells expressing CCR5 or α_4_β_7_, implying that HPV infection does not increase the risk of HIV acquisition via upregulation of CCR5 and α_4_β_7_ on cervical CD4 T cells. On the other hand, activated T cells are susceptible to HIV infection [57–59]; therefore activation of T cells as a result of HPV-associated lesions could increase the risk of acquiring HIV amongst HIV- women with HPV associated lesions. In contrast to HPV, we observed that HIV infection was associated with an inverted CD4/CD8 ratio and a decreased percentage of cervical T cells expressing CCR5 or α_4_β_7_. This suggests an HIV-driven depletion of these T cells from this anatomical compartment. Similar results have been reported for the gastrointestinal mucosa during acute and treated HIV infection, where CCR5+, as well as β_7_^Hi^ CD4 T cells were strongly and persistently depleted in HIV+ compared to HIV- individuals [60,61]. The exact mechanism by which these cells are depleted, whether by a direct cytopathic effect of HIV infection or bystander killing, is still unknown.

Chronic immune activation has been associated with exhaustion, senescence, and death of immune cells [62,63]. It has also been linked to a high risk of acquiring HIV infection [30,57], as well as to fast HIV disease progression [17]. Using a Rhesus monkey (*Macaca mulata*) Simian immunodeficiency virus (SIV) intravaginal infection model, Zhang et al., [49] have shown that HLA-DR+ cervical T cells are among the earliest cellular targets for SIV infection. In this study, these cells had a 4-fold increased levels of SIV RNA as compared to HLA-DR- cervical T cells, suggesting a critical role of immune activation of cervical T cells during early virus propagation that precedes systemic dissemination of AIDS virus infection. A phenotype of increased activation in HIV+ subjects has also been demonstrated for peripheral blood CD4 lymphocytes [64].

Our results show that HPV infection was associated with an increase in the proportion of HLA-DR+ total CD4 and CD4 T cells expressing HIV co-receptors CCR5 and α_4_β_7_. However, stratification by different permutations of HIV-HPV co-infection (HIV-HPV-, HIV-HPV+, HIV+HPV-, HIV+HPV+) did not show any statistical significance, possibly due to the current small sample size. Even though HPV infection was not associated with altered frequency of HIV co-receptors, the increase the in proportion of activated cervical CD4 T cells expressing HIV co-receptors could increase the risk of HIV acquisition among HIV- individuals [25,28, 55].

Furthermore, we observed an HIV-status independent increase in the percentage of HLA-DR+ T cells in women with HPV infections and especially cervical lesions. This indicates that both HPV infection and the associated lesions alter the activation of T cells in the cervical mucosa, and that such alterations could contribute to increased HIV susceptibility in HIV—women. The exact mechanisms by which HPV increases HIV acquisition are yet unknown since most studies examining this phenomenon are observational [25]. Liebenberg et al., [28],however, have shown that HPV infection is associated with a distinct cytokine profile, associated an elevated risk to acquire HIV. Plausibly, CD4 T cells are recruited to the cervix by these cytokines as a result of HPV infection, and associated pre-cancerous lesions become prime targets for HIV, thereby increasing the risk of HIV acquisition. In addition, our results suggest that HPV-associated lesions are by no means “immunological quiescent”. This is furthered supported by Papasavvas *et al*., who reported an increased frequency of activated CD8 T cells in peripheral blood as a result of a HR-HPV infection [65]. Future studies focussing on HPV-specific T cells could improve our understanding on the effect of HPV on HIV acquisition and shed more light on the impact of HIV on the HPV-specific immune response. Furthermore, exploring the influence of immune exhaustion on cervical T cells as a consequence of persistent HPV infection may lead to a better understanding of how cervical immune cells contribute to the combat of pathogens that infect the body via the cervical mucosa.

Similar to peripheral blood, we report elevated immune activation in cervical CD4 T cells from HIV+ when compared to HIV- women. Even though ART treatment partially restored CD4 T cell frequencies in HIV+ women, the level of immune activation in cervical T cells remained high despite ART, indicating ART might not fully reverse HIV-induced immune dysfunction. Moreover, the level of immune activation in cervical T cells was inversely correlated to peripheral blood absolute CD4 counts of the same individual. These results are in line with Jaspan *et al*., where HIV infection was associated with a high percentage of T cells expressing HLA-DR, CD38 and Ki67 in both cervical and peripheral T cells [66].

In conclusion, we show that HPV infection was not associated with an upregulation of HIV co-receptors on cervical CD4 T cells, therefore implying a different mechanism for a possible increased risk of HIV acquisition in HPV-infected women. Furthermore, the low frequency of cervical CD4 T cells associated with a higher state of immune activation in cervical T cells of HIV+ women despite ART treatment, compounded with the immune activation resulting from HPV and HPV induced lesions, might hinder efficient HPV clearance from the cervical mucosa and pave the way for cervical cancer.

## Supporting information

S1 Table Demographic and flow cytometric data. Study participant information: HIV status, ART status, HPV infection status and cervical lesion diagnosis. Flow cytometric data: Cell counts and percentages of cellular subsets expressing T cell memory and activation markers as well as HIV co-receptor molecules.
(XLSX)

S1 Fig Proportions of T cells and α_4_β_7_+ T cells in blood and cervix. **A**: Percentage of CD3+CD45+ cervical and peripheral T cells stratified by sample source. (n = 39). The frequency of CD3+CD45+ T cells is shown as a proportion off all events collected for each sample. Sample source is indicated on the x-axis. Statistical analysis was performed using the Wilcoxon matched-pairs signed ranks test. **B,C**: Percentage of α_4_β_7_+CD4+ and α_4_β_7_+CD8+ cervical and peripheral T cells stratified by sample source and HIV status. (n = 39, HIV- = 22 and HIV+ = 17). The frequency of α_4_β_7_+CD4+ (**B**) and α_4_β_7_+C8+ (**C**) T cells is shown as a proportion of CD3+CD45+ T cells for each sample. HIV status and sample source is indicated on the x-axis. Statistical analysis was performed using the Wilcoxon matched-pairs signed ranks test.
(TIF)

S2 Fig Percentage of CD4+ and CD8+ cervical T cells stratified by HIV status. (n = 468; HIV- = 253 and HIV+ = 215). The frequency of CD4+ (**A**) and C8+ (**B**) T cells is shown as a proportion of CD3+CD45+ T cells for each sample. HIV status is indicated on the x-axis. The median frequencies are indicated. Statistical analysis was performed using the Mann-Whitney U-test.
(TIF)

S3 Fig Frequencies of CCR5+, α_4_β_7_+_,_ or HLA-DR+ T cells stratified by HIV and ART. **A:** Cervical CD4 T cells proportions stratified by HIV and ART usage status (n = 454; HIV- = 253, HIV+ART+ = 153 and HIV+ART- = 48). The frequency of cervical CD4 T cells is shown as a percent of CD3+CD45+. Each dot represents one patient. HIV status and ART usage is indicated on the X axis. The median percentages are indicated. Statistical analysis was performed using the Mann-Whitney U-test. **B,C**: Percentage of CCR5+ and α_4_β_7_+ cervical CD4 T cells stratified by HIV and ART status (n = 454; HIV- = 253, HIV+ART+ = 153, HIV+ART- = 48) The frequency of CCR5+CD4+ (B) and α_4_β_7_+CD4+ (C) T cells is shown as a proportion of CD3+CD45+ T cells for each sample. HIV and ART status is indicated on the x-axis. The median frequencies are indicated. Statistical analysis was performed using the Mann-Whitney U-test. **D:** Percentage of HLA-DR+ CD4+ cervical T cells stratified by HIV and ART status. (n = 241; HIV- = 129, HIV+ART+ = 84, HIV+ART- = 28)The percentage of HLA-DR+ CD4+ T cells is shown as a proportion of CD3+CD45+ T cells for each sample. HIV and ART status is indicated on the x-axis. The median frequencies are indicated. Statistical analysis was performed using the Mann-Whitney U-test.
(TIF)

S4 Fig Frequencies of CCR5+, α_4_β_7_+_,_ or HLA-DR+ T cells stratified by HIV and HPV infection status. **A,B:** α_4_β_7_ and CCR5 frequencies on cervical CD4 T cells stratified by HIV and HPV infection status (n = 215; HIV+HPV+ = 75, HIV+HPV- = 25, HIV-HPV+ = 47, HIV-HPV- = 66). The frequency of α_4_β_7_+CD4+ (A) and CCR5+CD4+ (B) T cells is shown as a proportion of CD3+CD45+ T cells for each sample. HIV and HPV infections status is indicated on the x-axis. The median frequencies are indicated. Statistical analysis was performed using the Mann-Whitney U-test. **C,D:** Percentage of HLA-DR+ CD4 and CD8 cervical T cells stratified by HIV and HPV infection status. (n = 103; HIV+HPV+ = 40, HIV+HPV- = 6, HIV-HPV+ = 30, HIV-HPV- = 27). The percentage of HLA-DR+ CD4+ (C) and HLA-DR+ CD8+ T cells (D) is shown as a proportion of CD3+CD45+ T cells for each sample. HIV and HPV infections status is indicated on the x-axis. The median frequencies are indicated. Statistical analysis was performed using the Mann-Whitney U-test.
(TIF)

## Abbreviations

AIDS: Acquired Immunodeficiency Syndrome
ART: Antiretroviral therapy
CC: Cervical Cancer
CCR5: Chemokine receptor 5
FBS: Fetal Bovine Serum
HIV: Human Immunodeficiency Virus
HLA-DR: Human Leukocyte Antigen–DR isotype
HPV: Human Papillomavirus
HSIL: High-grade Squamous Intraepithelial Lesion
LSIL: Low-grade Squamous Intraepithelial Lesion
