# Supplementary figures and images for: Depletion and activation of mucosal CD4 T cells in HIV infected women with HPV-associated lesions of the cervix uteri
Source: PLoS One. 2020 Oct 2;15(10):e0240154. doi: 10.1371/journal.pone.0240154 (PMC7531815; doi:10.1371/journal.pone.0240154)

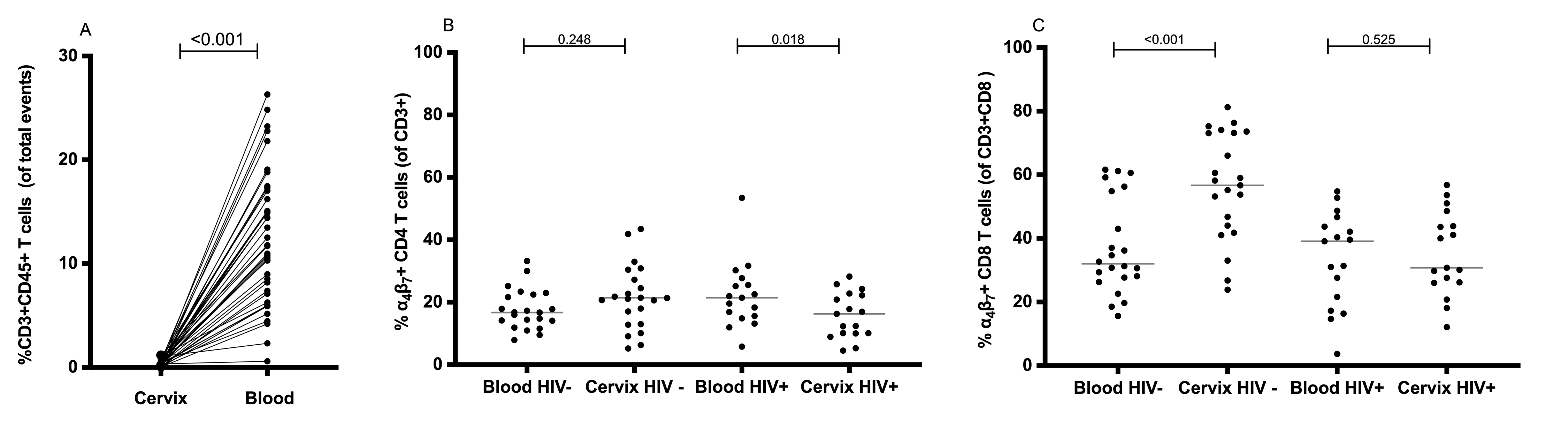

Supplement: S1 Fig — A: Percentage of CD3+CD45+ cervical and peripheral T cells stratified by sample source. (n = 39). The frequency of CD3+CD45+ T cells is shown as a proportion off all events collected for each sample. Sample source is indicated on the x-axis. Statistical analysis was performed using the Wilcoxon matched-pairs signed ranks test. B,C: Percentage of α4β7+CD4+ and α4β7+CD8+ cervical and peripheral T cells stratified by sample source and HIV status. (n = 39, HIV- = 22 and HIV+ = 17). The frequency of α4β7+CD4+ (B) and α4β7+C8+ (C) T cells is shown as a proportion of CD3+CD45+ T cells for each sample. HIV status and sample source is indicated on the x-axis. Statistical analysis was performed using the Wilcoxon matched-pairs signed ranks test. (TIF) [file pone.0240154.s002.tif]

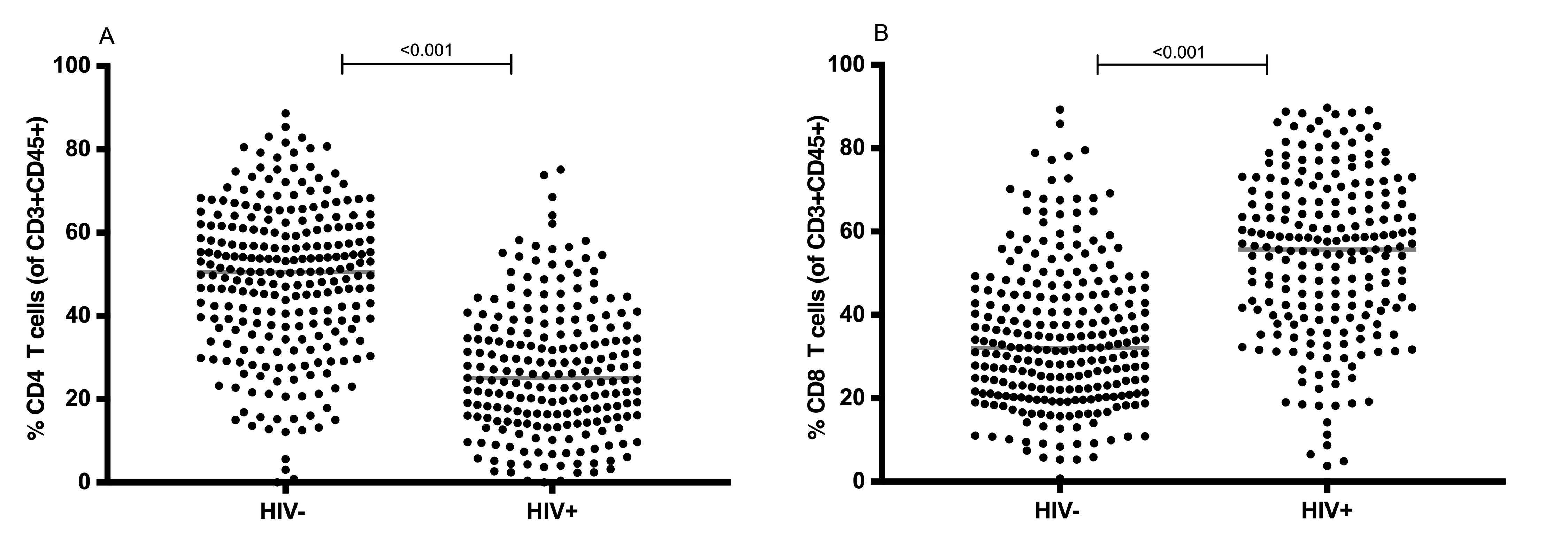

Supplement: S2 Fig — (n = 468; HIV- = 253 and HIV+ = 215). The frequency of CD4+ (A) and C8+ (B) T cells is shown as a proportion of CD3+CD45+ T cells for each sample. HIV status is indicated on the x-axis. The median frequencies are indicated. Statistical analysis was performed using the Mann-Whitney U-test. (TIF) [file pone.0240154.s003.tif]

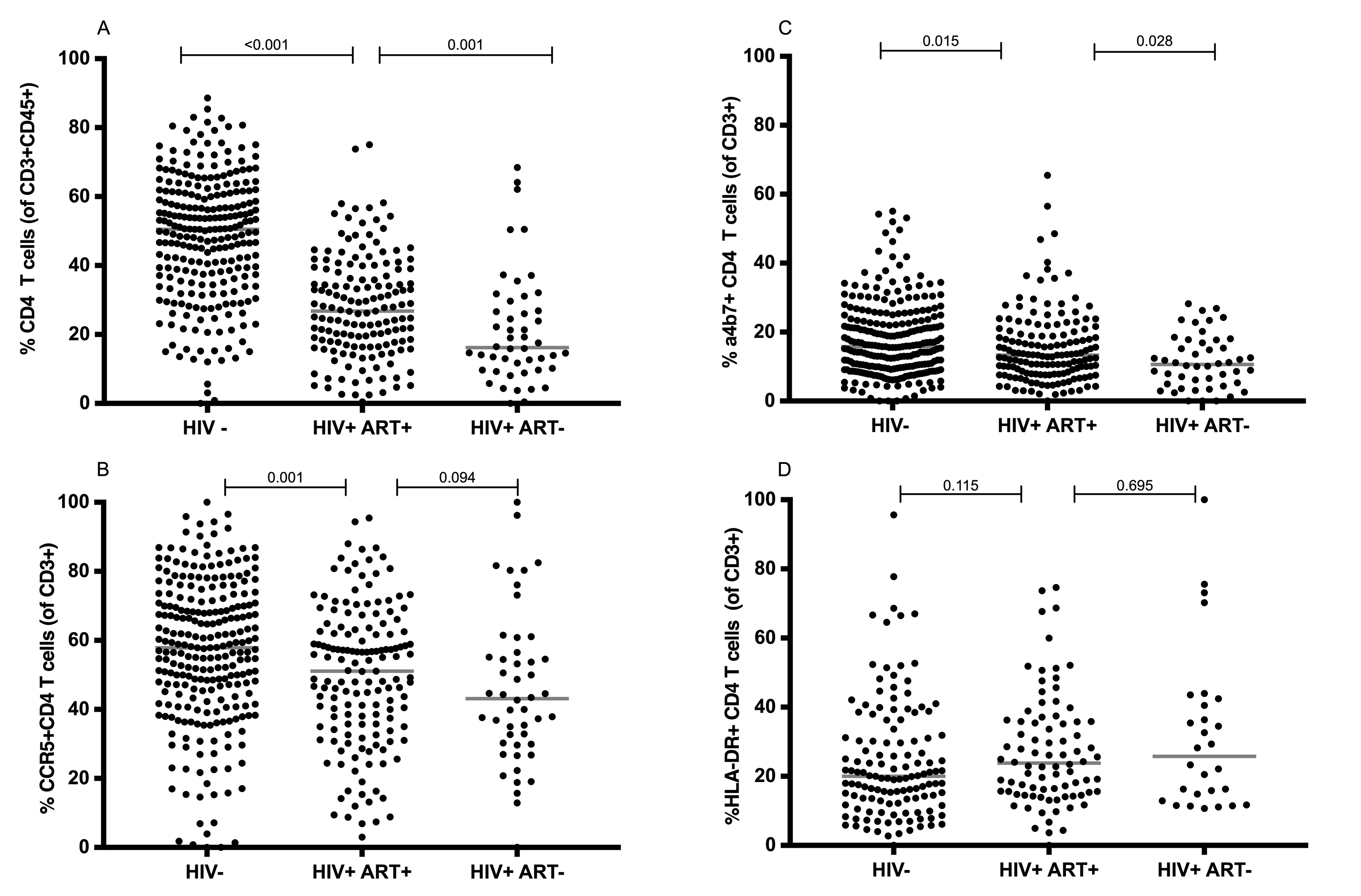

Supplement: S3 Fig — A: Cervical CD4 T cells proportions stratified by HIV and ART usage status (n = 454; HIV- = 253, HIV+ART+ = 153 and HIV+ART- = 48). The frequency of cervical CD4 T cells is shown as a percent of CD3+CD45+. Each dot represents one patient. HIV status and ART usage is indicated on the X axis. The median percentages are indicated. Statistical analysis was performed using the Mann-Whitney U-test. B,C: Percentage of CCR5+ and α4β7+ cervical CD4 T cells stratified by HIV and ART status (n = 454; HIV- = 253, HIV+ART+ = 153, HIV+ART- = 48) The frequency of CCR5+CD4+ (B) and α4β7+CD4+ (C) T cells is shown as a proportion of CD3+CD45+ T cells for each sample. HIV and ART status is indicated on the x-axis. The median frequencies are indicated. Statistical analysis was performed using the Mann-Whitney U-test. D: Percentage of HLA-DR+ CD4+ cervical T cells stratified by HIV and ART status. (n = 241; HIV- = 129, HIV+ART+ = 84, HIV+ART- = 28)The percentage of HLA-DR+ CD4+ T cells is shown as a proportion of CD3+CD45+ T cells for each sample. HIV and ART status is indicated on the x-axis. The median frequencies are indicated. Statistical analysis was performed using the Mann-Whitney U-test. (TIF) [file pone.0240154.s004.tif]

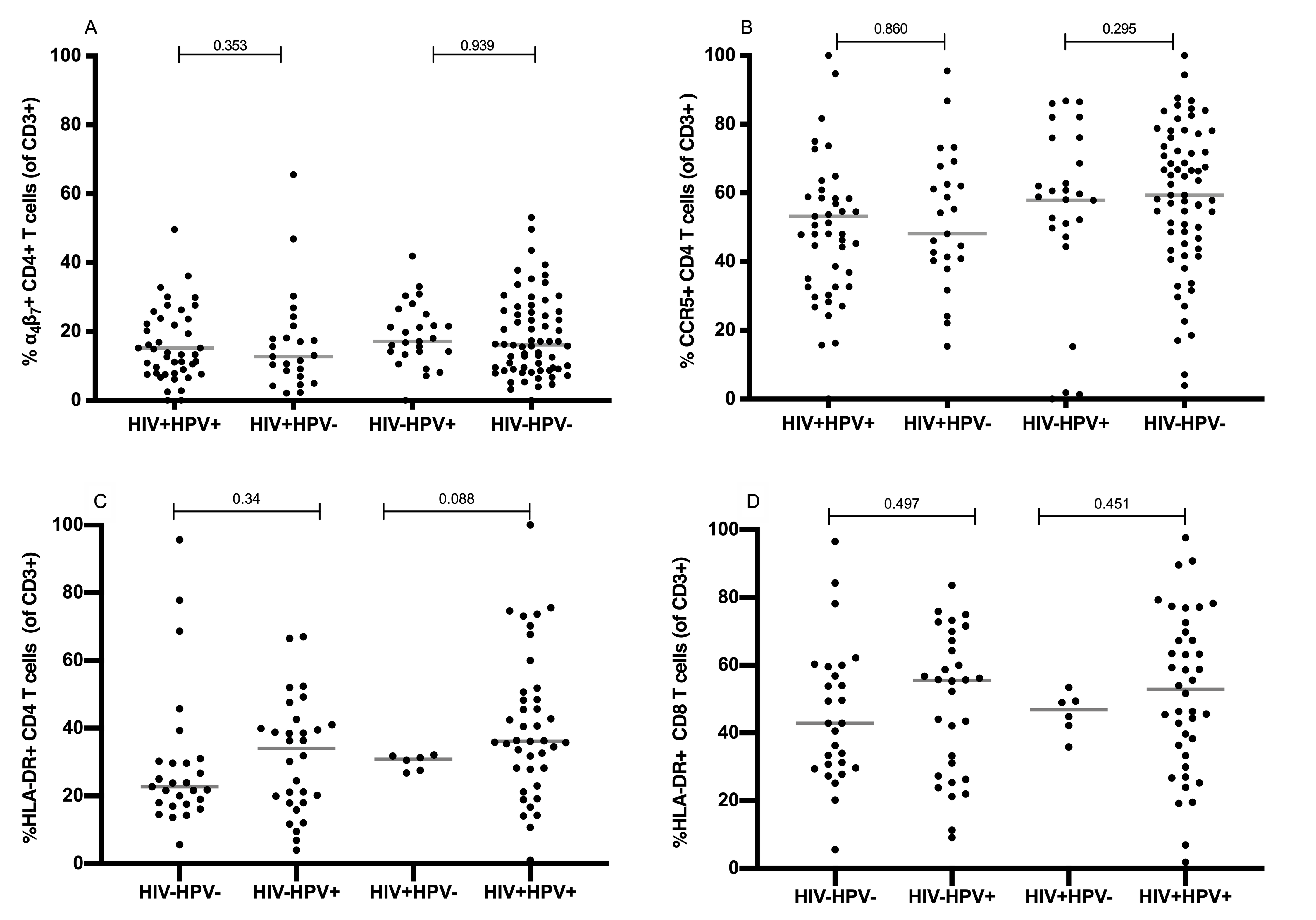

Supplement: S4 Fig — A,B: α4β7 and CCR5 frequencies on cervical CD4 T cells stratified by HIV and HPV infection status (n = 215; HIV+HPV+ = 75, HIV+HPV- = 25, HIV-HPV+ = 47, HIV-HPV- = 66). The frequency of α4β7+CD4+ (A) and CCR5+CD4+ (B) T cells is shown as a proportion of CD3+CD45+ T cells for each sample. HIV and HPV infections status is indicated on the x-axis. The median frequencies are indicated. Statistical analysis was performed using the Mann-Whitney U-test. C,D: Percentage of HLA-DR+ CD4 and CD8 cervical T cells stratified by HIV and HPV infection status. (n = 103; HIV+HPV+ = 40, HIV+HPV- = 6, HIV-HPV+ = 30, HIV-HPV- = 27). The percentage of HLA-DR+ CD4+ (C) and HLA-DR+ CD8+ T cells (D) is shown as a proportion of CD3+CD45+ T cells for each sample. HIV and HPV infections status is indicated on the x-axis. The median frequencies are indicated. Statistical analysis was performed using the Mann-Whitney U-test. (TIF) [file pone.0240154.s005.tif]
